# Development of a Novel Peptide with Antimicrobial and Mineralising Properties for Caries Management

**DOI:** 10.3390/pharmaceutics15112560

**Published:** 2023-10-31

**Authors:** Olivia Lili Zhang, John Yun Niu, Ollie Yiru Yu, May Lei Mei, Nicholas Stephen Jakubovics, Chun Hung Chu

**Affiliations:** 1 Faculty of Dentistry, The University of Hong Kong, Hong Kong 999077, China; zhlili@connect.hku.hk (O.L.Z.); niuyun@hku.hk (J.Y.N.); ollieyu@hku.hk (O.Y.Y.); may.mei@otago.ac.nz (M.L.M.); 2 Faculty of Dentistry, The University of Otago, Dunedin 9054, New Zealand; 3 School of Dental Sciences, Faculty of Medical Sciences, Newcastle University, Newcastle upon Tyne NE2 4BW, UK

**Keywords:** caries, prevention, mineralisation, peptides, antimicrobial

## Abstract

The purpose of the study is to develop a novel peptide for caries management. Gallic-Acid-Polyphemusin-I (GAPI) was synthesised by grafting Polyphemusin I (PI) and gallic acid (GA). Biocompatibility was evaluated using a Cell Counting Kit-8 Assay. Antimicrobial properties were assessed using minimum inhibitory concentration (MIC) and minimum bactericidal/fungicidal concentration (MBC/MFC). The bacterial and fungal morphology after GAPI treatment was investigated using transmission electron microscopy (TEM). The architecture of a consortium biofilm consisting of *Streptococcus mutans, Lacticaseibacillus casei* and *Candida albicans* was evaluated using scanning electron microscopy (SEM) and confocal laser scanning microscopy. The growth kinetics of the biofilm was examined using a propidium monoazide–quantitative polymerase chain reaction. The surface and calcium-to-phosphorus molar ratio of GAPI-treated enamel after pH cycling were examined with SEM and energy-dispersive X-ray spectroscopy. Enamel crystal characteristics were analysed using X-ray diffraction. Lesion depths representing the enamel’s mineral loss were assessed using micro-computed tomography. The MIC of GAPI against *S. mutans*, *L. casei* and *C. albicans* were 40 μM, 40 μM and 20 μM, respectively. GAPI destroyed the biofilm’s three-dimensional structure and inhibited the growth of the biofilm. SEM showed that enamel treated with GAPI had a relatively smooth surface compared to that treated with water. The calcium-to-phosphorus molar ratio of enamel treated with GAPI was higher than that of the control. The lesion depths and mineral loss of the GAPI-treated enamel were less than the control. The crystallinity of the GAPI-treated enamel was higher than the control. This study developed a biocompatible, mineralising and antimicrobial peptide GAPI, which may have potential as an anti-caries agent.

## 1. Introduction

Antimicrobial peptides are naturally occurring antimicrobial molecules produced by multicellular or unicellular organisms [1]. Antimicrobial peptides are potential alternatives to traditional antimicrobial agents for use as therapeutic agents [2]. The typical secondary structures of antimicrobial peptides are α-helical, β-sheet, loop and extended structures [3]. The interaction of antimicrobial peptides with the cytoplasmic membrane results in increased membrane permeability and finally leads to membrane lysis and cell content release [4].

Natural antimicrobial peptides derived from diverse sources have inspired researchers to develop synthetic peptides [5]. In addition, the flexible structure of antimicrobial peptides allows various functional modifications which extend their potential applications [5]. Fused peptides with antimicrobial and functional domains is a workable strategy for developing novel peptides for caries management [2]. For instance, a specific target *S. mutans* peptide C16G2 was synthesised by the fusion of broad-spectrum antimicrobial peptide G2 and selectively targeted peptide domain C16 [6,7].

In the oral cavity, the role of antimicrobial peptides is critical for controlling multiple oral diseases, including dental caries [4]. Dental caries is one of the most common chronic diseases worldwide. It results from mineral loss due to the interaction of basic calcium phosphate with protons (hydrogen ions) [8]. The hydrogen ions come from organic acids produced by microbial fermentation within dental biofilms [9]. 

Although many different species contribute to the cariogenicity of biofilms, certain lactic acid producing bacteria such as *S. mutans* and *L. casei* have received particular attention due to their high capacity for acid production from free sugars [10]. *S. mutans* also synthesizes insoluble glucan polymers from sucrose, which is thought to further contribute to caries activity [11]. In addition, fungi such as *C. albicans* are implicated in caries formation, particularly in early childhood caries (ECC) [12]. Given the diverse species and functional modifications of antimicrobial peptides, it is possible to develop an anti-caries peptide with both antimicrobial and mineralising properties.

Polyphemusin-I (PI) is an antimicrobial peptide derived from horseshoe crabs showing antimicrobial properties against *S. mutans* [13]. Gallic acid (GA) is a naturally abundant molecule that can be extracted from *Galla chinensis*, one of the important Chinese herbs [14,15]. Gallic acid has been recognized as a potential inhibitor of demineralisation [16]. Cheng et al. reported GA-facilitated mineral deposition predominately on the surface layer of demineralised enamel [17]. 

The proposed mechanism involves the pyrogallol moiety of GA, which can strongly bind to calcium ions and then accelerate regeneration of hydroxyapatite [18,19]. The conjugation method to develop fused peptides with antimicrobial peptides and functional domains is a workable strategy for developing novel peptides for caries management [4]. Thus, the aim of this study is to develop a novel peptide (GAPI) using antimicrobial domain PI and mineralising domain GA. We hypothesised that the novel peptide will have a dual function of antimicrobial and mineralising properties.

## 2. Materials and Methods

### 2.1. Synthesis and Characterisation

A novel peptide, GAPI, was designed by fusing GA to the N-terminus of PI (RRWCFRVCYRGFCYRKCR). PI and GAPI were synthesised using the solid-phase peptide synthesis metho [20]. The synthesised peptides underwent lyophilisation for preservation. The purity and molecular weight of peptides were determined using high-performance liquid chromatography (HPLC) and mass spectrometry. The secondary structure of peptides was investigated using circular dichroism spectroscopy (Chirascan, AppliedPhotophysics, Leatherhead, UK) and modelled using the software CDPro (https://sites.google.com/view/sreerama, 13 January 2022) [21].

### 2.2. Stability in Human Saliva

The stability of peptides in human saliva (collected from six individual healthy volunteers; three males and three females; minimum age, 29; and maximum age, 36) was investigated by testing the remaining peptide concentration via HPLC system (Waters Pacific Pte Ltd., Singapore). The test was processed following a previous experimental protocol [22]. After centrifuging the saliva and collecting the supernatant, GAPI was added to the sample and incubated at 37 °C for 60 min. A 1 mL sample was taken and analysed using HPLC system.

### 2.3. Biocompatibility

The biocompatibility of GAPI was evaluated by cytotoxicity assay with human gingival fibroblast (HGF-1, Otwo Biotech Inc., Shenzhen, China) cells (1 × 10^5^/mL). The Cell Counting Kit-8 assay (CCK-8, Apexbio, Houston, TX, USA) was conducted [13].

### 2.4. Antimicrobial Properties

The antimicrobial properties of synthesised peptides against bacteria and fungus were evaluated using minimum inhibitory concentration (MIC) and minimum bactericidal/fungicidal concentration (MBC/MFC) tests [23]. The tested species include *S. mutans*, UA159; *L. casei,* American Type Culture Collection (ATCC) 334; and *C. albicans*, ATCC 90028. A 10 μL bacterial culture (10^6^ CFU/mL) in brain heart infusion (BHI) broth was added to 100 μL serial two-fold dilutions of experimental solutions. The optical density values at 660 nm were measured after 18 h of incubation. To determine the MBC, 10 μL of bacterial culture was taken and transferred to blood agar. The test protocol for fungus was similar: the culture medium was a Roswell Park Memorial Institute (RPMI) 1640 Medium, the absorbance was measured at 520 nm, and Sabouraud dextrose agar was used for the MFC test. Each experiment was performed in triplicate.

The morphology of the bacteria and fungus treated with synthesised peptides was analysed using transmission electron microscopy (TEM) (Philips CM100, Philips/FEI Corporation, Eindhoven, North Brabant, The Netherlands) [21].

### 2.5. Antibiofilm Properties

A multiple-species cariogenic biofilm consisting of *S. mutans*, *L. casei* and *C. albicans* was cultured following a previous protocol [24]. The architecture of the multiple-species biofilm with or without GAPI treatment was assessed using scanning electron microscopy (SEM) and confocal laser scanning microscopy (CLSM) [21]. Propidium iodide and 6-diamidino-2-phenylindole (DAPI), which highlight bacterial cells, and Concanavalin A (ConA), which labels *C. albicans*, were used to stain the multiple-species biofilm. The growth kinetics of the multiple-species biofilm were analysed using propidium monoazide–quantitative polymerase chain reaction (PMA-qPCR) (Supplementary Material S1).

### 2.6. Mineralising Effects

A total of 40 enamel blocks were prepared from extracted sound human molars. The pH-cycling procedure (16 h demineralisation at pH 4.5 and 8 h remineralisation at pH 7.0) was performed on enamel blocks for 8 days, using solutions that contained calcium and phosphate [21]. The enamel blocks from the same tooth were treated twice daily with 160 μM GAPI (GAPI Group), 160 μM GA (GA Group), 160 μM PI (PI Group) and sterile deionised water (Water Group). The structure of the enamel surface was examined using SEM (*n* = 8 per group) [21]. The elemental analysis of enamel surface was conducted using affiliated energy-dispersive X-ray spectroscopy (EDS). The calcium-to-phosphorus molar ratio was calculated [21]. 

The mineral loss and lesion depth on the enamel blocks were assessed using microcomputed tomography (Micro-CT) (1172, SkyScan, Antwerp, Belgium) (*n* = 8 per group) [20]. The parameters of Micro-CT were 80 kV voltage and 100 μA current with a voxel size of 12 μm^3^. The image was reconstructed with NRecon software 1.7.4.6 (SkyScan, Antwerp, Belgium) and analysed using CTAn 1.20.3.0 (SkyScan, Antwerp, Belgium) to determine the lesion depth and mineral density values (MDVs, gHApcm^−3^). The mineral loss = MDV _of the control area_ − MDV _of the demineralised area_.

The diffraction patterns of the enamel blocks were evaluated using X-ray diffraction analysis (XRD) (Rigaku SmartLab 9 kW, Bruker AXS GmbH, Karlsruhe, Germany) (*n* = 2 per group) [20].

### 2.7. Sample Size Calculation and Statistical Analyses

The sample size was estimated using G*Power software 3.1 [25]. The mean lesion depth of the experimental group was 60 μm in our pilot study. Assuming that the largest difference was 25 μm and common standard deviation was 12 μm (which means the coefficient of variation estimated at around 20%) with power at 0.9 and α = 0.05, the sample size was at least six in each group. Thus, we set the sample size at *n* = 8. The data were analysed using SPSS Statistics 20 (IBM Corporation, Somers, NY, USA).

## 3. Results

The purity percentage of GAPI was 96.74% (Supplementary Material S2). The molecular weight was 2608.05 kD for GAPI (Supplementary Material S3). The secondary structure analysis of GAPI showed that the proportion of the β-sheet was 48.0% (Supplementary Material S4). The remaining percentage of GAPI after incubation in human saliva for 60 min was 94.4%. There was no significant difference in optical density values between the 640 μM GAPI-treated HGF-1 cells and the negative control cell (Supplementary Material S5).

### 3.1. Antimicrobial Properties

**Figure 1:**
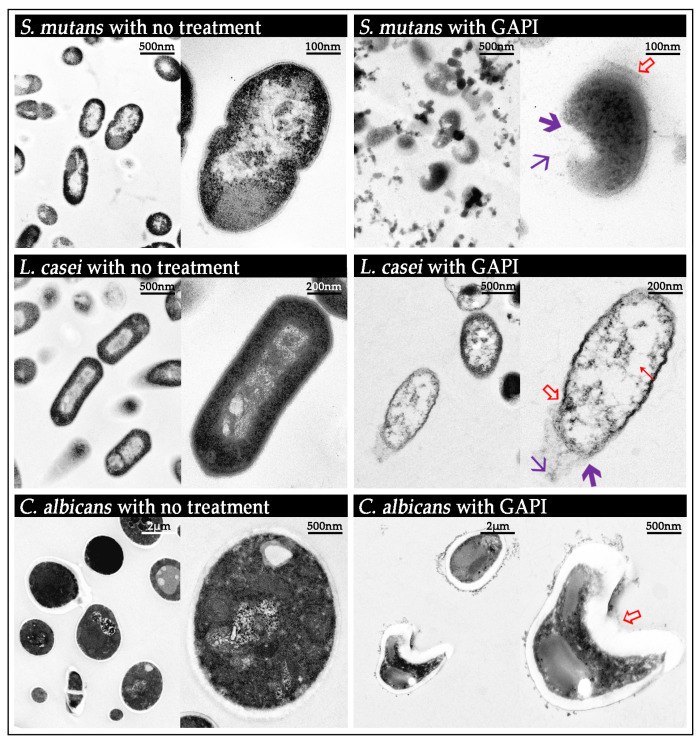
TEM micrographs of the bacteria and the fungus before and after GAPI treatment. 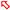 Abnormal cell morphology; **↖** cytoplasmic clear zone; 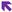 disrupted membrane/cell wall; and 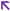 cytoplasmic content leakage.

The morphology of microorganisms treated with GAPI displayed severe defects in TEM micrographs (Figure 1). *S. mutans* treated with GAPI exhibited abnormal cell membrane curvature and a disrupted cytoplasmic membrane with leakage of cytoplasmic contents. *L. casei* treated with GAPI mainly exhibited an irregular cell shape and prominent cytoplasmic clear zones. As for GAPI-treated *C. albicans*, the primary defect was abnormal cell membrane curvature and an irregular cell shape.

MIC and MBC/MFC values of GAPI and PI against tested bacteria and fungus are shown in Table 1.

### 3.2. Antibiofilm Properties

**Figure 2:**
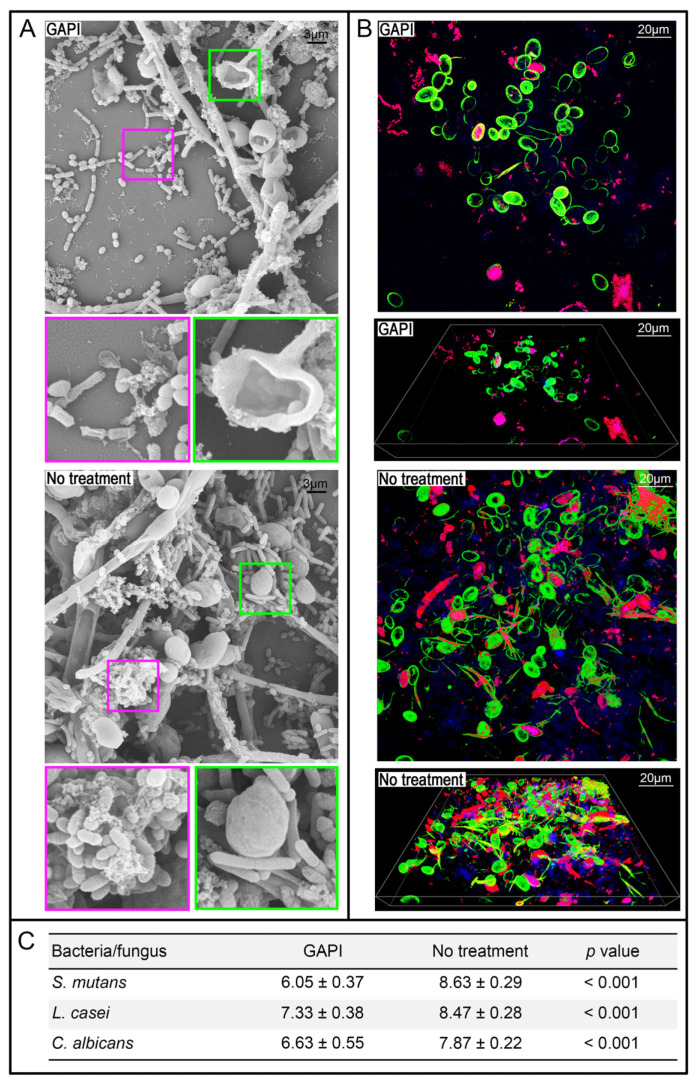
Three-species biofilm with or without GAPI peptide treatment. (**A**) Scanning electron micrographs of the biofilm with or without GAPI treatment. GAPI treatment inhibited biofilm formation and altered cell morphology. (**B**) Confocal laser scanning micrographs of the biofilm with or without GAPI treatment. GAPI reduced the microorganisms. *C. albicans* (green) could be distinguished from bacterial cells (pink). (**C**) Log10 cell counts/biofilm of the bacteria/fungus in a three-species biofilm with or without GAPI peptide (*n* = 6 per group).

Figure 2 shows the SEM micrographs and CLSM photographs of a multiple-species biofilm treated with or without GAPI.

The GAPI treatment inhibited the forming of a biofilm. In addition, the SEM micrographs showed that the cells in the biofilm treated with GAPI lost their typical morphology (Figure 2A). In contrast, a three-dimensional multiple-species biofilm formed in the control group. 

The CLSM photographs also demonstrated that GAPI obviously reduced the fluorescent signal of microorganisms in the biofilm (Figure 2B). The log10 cell counts/biofilm of three species treated with GAPI were significantly lower than those of untreated species (Figure 2C).

### 3.3. Mineralising Effects

**Figure 3:**
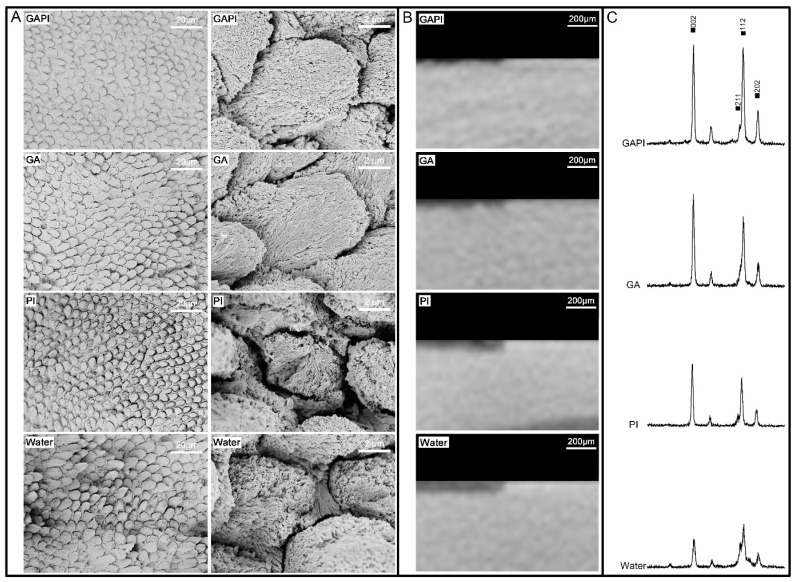
Enamel treated with 160 μM GAPI, 160 μM GA, 160 μM PI or deionised water (Water). (**A**) Scanning electron micrographs of the enamel in four groups. (**B**) Microcomputed tomographs of the enamel in four groups. (**C**) X-ray diffraction patterns of the enamel in four groups.

The SEM micrographs of the enamel surface showed a relatively smooth and homogenous prism pattern in the GAPI Group and the GA Group compared to other groups (Figure 3A). In addition, newly formed particles could be observed on the enamel surface. In the PI Group and Water Group, the destruction of enamel prisms occurred in both the prism and inter-prism regions (Figure 3A).

Moreover, the enamel in the GAPI Group and GA Group had significantly higher calcium-to-phosphorus molar ratios than that in other groups (Table 2).

The Micro-CT figures show a consistent result compared to SEM micrographs: a damaged surface in the PI Group and the Water Group but a relatively undamaged enamel surface in the GAPI Group and the GA Group (Figure 3B). In addition, the enamel in the GAPI Group and the GA Group had significantly lower lesion depths and less mineral loss than those of the enamel in the PI Group and Water Group (Table 2).

Figure 3C shows the typical X-ray diffraction spectra of the four groups. The diffraction peaks at 25.9°, 31.8°, 32.2° and 34.0° represent reflections of (002), (211), (112) and (202), respectively, for hydroxyapatite. The reflections of hydroxyapatite in the GAPI Group and GA Group were sharper compared with the other groups. The full width at half maximum of reflection peak for the four groups were 0.237°, 0.231°, 0.342° and 0.328°.

## 4. Discussion

This is the first study to develop an antimicrobial peptide with mineralising properties by fusing the mineralising action domain, GA and the antimicrobial action domain, PI. The results showed that GAPI inhibited the growth of cariogenic bacteria/fungus and mineralised early enamel lesions. These results further confirmed that the conjugation method is an effective strategy for developing novel peptides for caries management. Researchers will discover more novel peptides with the application of this method. They will have opportunities to identify excellent peptides for clinical caries management in the near future.

The first step for synthesising the novel peptide GAPI is the production of PI domain by bonding the carboxyl group (C terminus) of one amino acid to the amino group (N terminus) of another amino acid. The peptide PI could be extracted and purified from horseshoe crabs [26]. The synthesis method of GAPI used the standard fluorenylmethoxycarbonyl solid-phase synthesis method, which is the method of choice for peptide synthesis and can guarantee a high purity of the synthesised peptides. Although it is currently cost-prohibitive to produce peptides on the scale required for routine oral hygiene, Behrendt et al. predicted a decline in the cost of the method because of advances in technology and the economies of scale arising from the large-scale production of therapeutic peptides [27].

The secondary structure of peptides was measured using circular dichroism spectroscopy. PI displays a β-turn structure with connected antiparallel β-sheets. The β-sheet antimicrobial peptides can bind to the lipid bilayer of microbial membranes, electrostatically interact with the bacterial cell and translocate across the lipid bilayer. These activities cause the disruption of the cytoplasmic membrane, which is a possible antimicrobial mechanism of peptides [28]. In our study, the percentages of the β-sheet of the GAPI were lower than those of PI, which may explain why the antimicrobial activity of GAPI is slightly less than PI alone. However, this minor reduction is acceptable because we demonstrate the novel peptide’s mineralisation ability in this study.

The biocompatibility and stability of the synthesised peptide is vitally important for further use in the dental clinic. The results showed that the concentration of GAPI starting to affect the proliferation of a human gingival fibroblast is higher than its minimum bactericidal/fungicidal concentration against cariogenic species. These results suggested the low toxicity of GAPI for human cells. Thus, GAPI is biocompatible with human gingival fibroblasts. Further confirmation of GAPI’s safety for dental use is necessary through testing in animal models before human clinical trials can be considered. 

Human saliva maintains a neutral condition because of its buffering activity and continual replenishment. Our study showed that GAPI was stable for at least 60 min in saliva. Due to the flowing nature of the human oral environment, the efficiency of dental materials can be influenced by saliva washout [29]. Therefore, it is crucial for the materials to have effective concentration and stable properties. In addition, multiple active ingredients may interact with one another and affect the stability of the product [30]. Hence, reducing the number of active components is desirable. We combined GA and PI to a novel stable agent. It has established a strong foundation for clinical translation.

We assessed the antimicrobial effect of peptides against bacteria/fungus under planktonic conditions. Many lines of evidence indicate that *S. mutans* is an important species in caries development. It can adhere to the acquired enamel pellicle and other oral bacteria, and most strains have traits that are associated with dental caries such as strong acidogenicity and aciduricity, and the capacity to produce exopolysaccharides from sucrose [21]. *L. casei* is frequently detected in caries lesions and is associated with dental caries [10]. *C. albicans* also contributes to caries progression or recurrence [31]. In our study, GAPI inhibited planktonic *S. mutans*, *L. casei* and *C. albicans*. However, the MIC and MBC/MFC values of GAPI are higher than those of PI, particularly against *C. albicans*.

In addition, we analysed the cell morphology of bacteria/fungus treated with GAPI to investigate the potential mechanism of antimicrobial activity. An abnormal membrane curvature was the most typical defect. Moreover, it is likely that the peptide translocates across the lipid bilayer to act on intracellular targets to form transparent cytoplasmic zones. These results are consistent with descriptions of the antimicrobial mechanisms of the peptides in other studies [13]. 

We also found that a thorough disruption of the cytoplasmic membrane occurred in *S. mutans* cells, which can cause leakage of the cytoplasmic content. Previous studies indicated that β-sheet peptides, such as PI, are not likely to induce pore formation or significant membrane damage [32]. However, the different membrane-active mechanisms should not be mutually exclusive; they may be mutually causal [33]. In addition, this is the first study to investigate the damage of a peptide derived from PI on *S. mutans* by using TEM. These findings brought new knowledge on the mechanism of antimicrobial peptides. Overall, our data indicated that GAPI has antimicrobial properties similar to other β-sheet antimicrobial peptides.

We used *S. mutans*, *L. casei* and *C. albicans* to develop a multiple-species cariogenic biofilm for assessing the anti-biofilm properties of GAPI. We used ConA to examine *C. albicans* and the bacteria, which could be clearly distinguished under CLSM [24]. In addition, the quantitative analysis of microorganisms was conducted via PMA-qPCR. A real-time PCR method combined with PMA was conducted [34] because discriminating live and dead cells is essential to assess the activity of antimicrobial peptides. 

The SEM, CLSM and PMA-qPCR results jointly demonstrated the anti-biofilm effect of GAPI on multiple-species biofilms. The reductions for the three species were more than 10-fold. A reduction in the number of cariogenic species is a positive development for caries prevention. In addition, a multiple-species cariogenic biofilm is superior compared to a mono-species biofilm for studying caries activity [35]. However, the results cannot be extrapolated because around 700 species have been shown to be capable of colonising the oral cavity [36]. 

A possible alternative would be to use a biofilm model inoculated with an oral microcosm. There are challenges with reproducibility in microcosm models due to their inherent complexity and the decreased microbial diversity that arises during in vitro culture [37]. Nevertheless, it would be useful to assess the effects of GAPI on cariogenicity in more complex biofilm models in future.

A chemical pH-cycling model was used to investigate the mineralising effect of GAPI in this study. This model uses a chemical acid challenge to simulate a high-risk caries situation. 

In addition, this model used a neutral solution saturated with calcium and phosphate to mimic saliva for mineralisation [38]. However, other components of saliva such as mucins that could impact de- and re-mineralisation were not included [39]. Future work should assess cariogenicity in the presence of organic components of saliva, as well as in different levels of fluoride to reflect oral hygiene practices more closely.

After the pH cycling, a non-destructive measurement, Micro-CT, was used to assess the enamel blocks. The advantages of Micro-CT include avoiding the need for a complicated sample preparation. The samples can still be used for other tests after scanning and the collected data could be transferred to quantifiable data (lesion depth and mineral loss) for statistical analysis. Nonetheless, a manual measurement on a computer monitor could introduce bias. We used sound enamel for the acid challenge and found that the control (water) group had a larger lesion depth and more mineral loss than the experimental (GAPI) group.

Furthermore, SEM-EDS was used to analyse the elemental composition of the enamel block’s surface. Then, the Ca/P molar ratio, which is related to the solubility of calcium phosphate compounds, could be calculated. In general, the higher the Ca/P molar ratio, the less soluble the calcium phosphate compounds [40]. The Ca/P molar ratios for tricalcium phosphate and less soluble fluorapatite were 1.50 and 1.67 [40]. However, EDS cannot identify the form of calcium and its precipitation affects the Ca/P molar ratio. The Ca/P molar ratio in GA-containing groups are higher than those without GA because GA is a calcium binder. 

We used X-ray diffraction analysis to study the hydroxyapatite crystallinity in the enamel blocks and assessed the full-width half-maximum value. The GA-containing groups had relatively low full-width half-maximum values indicating that they had a higher crystallinity (larger crystal size) than the other groups [41]. This result suggested that GAPI facilitated mineralisation. A possible mechanism for GA’s promotion of mineralization may involve its pyrogallol moiety, which consists of three hydroxyl groups with chelating abilities [18,19]. These hydroxyl groups exhibit a high affinity for binding with metal ions, such as calcium. When GA interacts with calcium ions, it forms a complex through chelation, subsequently interacting with phosphate ions. This coordination results in the nucleation of hydroxyapatite crystals. However, the precise molecular mechanisms underlying the interaction between GA, calcium and phosphate ions and the specific details of crystal nucleation and growth remain unclear. Further research is necessary to elucidate the role of the GA domain in GAPI for promoting mineralization.

For the descriptive analysis, an SEM micrograph confirmed the enamel surface structure. Sound enamel should have a smooth surface in the SEM micrograph. At the beginning of demineralisation, the prism patterns resembled well-organised fish scales due to the enamel crystal dissolution from the prisms’ edge. After a prolonged demineralisation, the dissolution of the enamel crystal occurred in both the prism and inter-prism regions. The enamel surface was uneven, and the prism patterns were disordered. We observed consistent results among the statistical and descriptive analyses, which illustrate the mineralising properties of GAPI. Since we used sound enamel in this study, a further study using an artificial caries model should be performed to confirm the remineralising effects of GAPI. This is a pilot study of the novel peptide GAPI. Further studies are essential to demonstrate GAPI’s antimicrobial and remineralising properties.

## 5. Conclusions

This laboratory study combined an antimicrobial domain PI and a mineralising domain GA to develop the biocompatible and stable peptide GAPI. The peptide inhibited the growth of cariogenic bacteria and fungus in planktonic conditions. In addition, it inhibited the growth of a multiple-species biofilm consisting of *S. mutans*, *L. casei* and *C. albicans*. It also reduced the demineralisation of early enamel carious lesions. This novel peptide has both antimicrobial and mineralising properties. Further animal studies are necessary to assess the anti-caries efficacy of GAPI. 

## 6. Patents

The work reported in this manuscript has been submitted for US Provisional Patent Application No.63/582,649 filed on 14 Sept 2023 and US Provisional Patent Application No.63/584,538 filed on 22 Sept 2023.

## Figures and Tables

**Table 1:** MIC and MBC/MFC of the two peptides.

Bacteria/Fungus	GAPI		PI	
	MIC (μM)	MBC/MFC (μM)	MIC (μM)	MBC/MFC (μM)
*S. mutans*	40	80	20	80
*L. casei*	40	160	20	160
*C. albicans*	20	40	0.3	0.6

**Table 2:** Mineral evaluations of sound enamel treated with GAPI, GA, PI and deionised water (Water) after pH cycling (*n* = 8 per group, mean ± SD). The number means four group in the Bonferroni line.

	GAPI ^1^	GA ^2^	PI ^3^	Water ^4^	*p* Value	Bonferroni
Calcium-to-phosphate molar ratio	1.81 ± 0.05	1.82 ± 0.08	1.70 ± 0.06	1.71 ± 0.05	=0.001	1, 2 > 3, 4
Lesion depth (µm)	64 ± 7	62 ± 12	96 ± 9	100 ± 10	<0.001	1, 2 < 3, 4
Mineral loss (gHApcm^−3^)	0.89 ± 0.20	0.88 ± 0.24	1.32 ± 0.26	1.28 ± 0.16	<0.001	1, 2 < 3, 4

## Data Availability

Data are contained within the article.

## Supplementary Materials

The following supporting information can be downloaded at: https://www.mdpi.com/article/10.3390/pharmaceutics15112560/s1, Supplementary Material S1. Experimental protocol: Propidium monoazide–quantitative polymerase chain reaction (PMA-qPCR). Supplementary Material S2. Figure: High-performance liquid chromatography for Gallic-Acid-Polyphemusin-I (GAPI) and Polyphemusin-I. Supplementary Material S3. Figure: Mass spectrometry analysis for Gallic-Acid-Polyphemusin-I (GAPI) and Polyphemusin-I. Supplementary Material S4. Figure: Circular dichroism spectroscopy spectrum for for Gallic-Acid-Polyphemusin-I (GAPI) and Polyphemusin-I. Supplementary Material S5. Figure: Concentration of Gallic-Acid-Polyphemusin-I (GAPI) and optical density values of human gingival fibroblasts.
