# Supplementary material for: Development of a Novel Peptide with Antimicrobial and Mineralising Properties for Caries Management
Source: Pharmaceutics. 2023 Oct 31;15(11):2560. doi: 10.3390/pharmaceutics15112560 (PMC10675526; doi:10.3390/pharmaceutics15112560)
Supplement: Supplementary file 1 [file pharmaceutics-15-02560-s001.zip › pharmaceutics-2603039-supplementary.pdf]

Cover Page

Supplementary S1 Experimental protocol: Propidium monoazide–quantitative polymerase chain reaction (PMA-qPCR) ……………………………………………………………………………..1

Supplementary S2 Figure: High-performance liquid chromatography for Gallic-Acid-Polyphemusin-I (GAPI) and Polyphemusin-I ………………………………………………………………….2

Supplementary S3 Figure: Mass spectrometry analysis for Gallic-Acid-Polyphemusin-I (GAPI) and Polyphemusin-I………………………………………………………………………………3

Supplementary S4 Figure: Circular dichroism spectroscopy spectrum for for Gallic-Acid-Polyphemusin-I (GAPI) and Polyphemusin-I………………………………………………………4

Supplementary S5 Figure: Concentration of Gallic-Acid-Polyphemusin-I (GAPI) and optical density values of human gingival fibroblasts………………………………………………………………5

Supplementary S1
Experimental protocol. Propidium monoazide–quantitative polymerase chain reaction (PMA-qPCR) test

The growth kinetics of the multiple-species biofilm were analysed using propidium monoazide–quantitative polymerase chain reaction (PMA-qPCR). Biofilm was suspended in phosphate-buffered solution. The suspension was treated with propidium monoazide (PMA) (Biotium, San Francisco, USA) and exposed to halogen light photoactivation (PMA-Lite™, Biotium, San Francisco, USA). The brightness (luminosity) of the light device is 600-800 millicandela and its wavelength is 465-475 nm. Then deoxyribonucleic acid (DNA) was extracted from PMA-treated biofilm suspension using QIAamp DNA Mini Kit (QIAGEN, Düsseldorf, Germany). The oligonucleotide primers and TaqMan probes were used to quantify multiple-species cariogenic biofilm species: SMUT-forward(F), SMUT-reverse(R) and SMUT-probe(P) for *Streptococcus mutans*, Lc-F, Lc-R, Lc-P for *Lactobacillus casei* and Ca-F, Ca-R and Ca-P for *Candida albicans* (**Table**). Taqman reactions contained 1μl each primer and probe, 10μl master mix (Applied Biosystems, Waltham, USA), 2μl sterile water and 5μl sample DNA. The thermocycling was conducted in a StepOnePlus real-time PCR system (Applied Biosystems, Waltham, USA) under the following conditions: an initial 2 min at 50 ℃, followed by a denaturation step for 10 min at 95℃, followed by 50 cycles of 95℃ for 15 s and 58℃ for 60 s. A standard curve was constructed using a series of known concentrations of microorganism cells for each species.

Table. List of the oligonucleotide primers and TaqMan probes for the propidium monoazide–quantitative polymerase chain reaction test

Name	Sequence
Ca-F	5′-GTGAATCATCGAATCTTTGAAC-3′
Ca-R	5′-TCCTCCGCTTATTGATATGC3′
Ca-P	6FAM-ATTGCTTGCGGCGGTAACGTCC-TAMRA
Lc-F	5′-AGGCGGCTGTCTGGTCTGTA-3′
Lc-R	5′-CCTGTTCGCTACCCATGCTT-3′
Lc-P	6FAM-TGACGCTGAGGCTC-TAMRA
SMUT-F	5′-GCCTACAGCTCAGAGATGCTATTCT-3′
SMUT-R	5′-GCCATACACCACTCATGAATTGA-3′
SMUT-P	6FAM- TGGAAATGACGGTCGCCGTTATGAA-TAMRA

Gallic-Acid-Polyphemusin-I Chromatogram

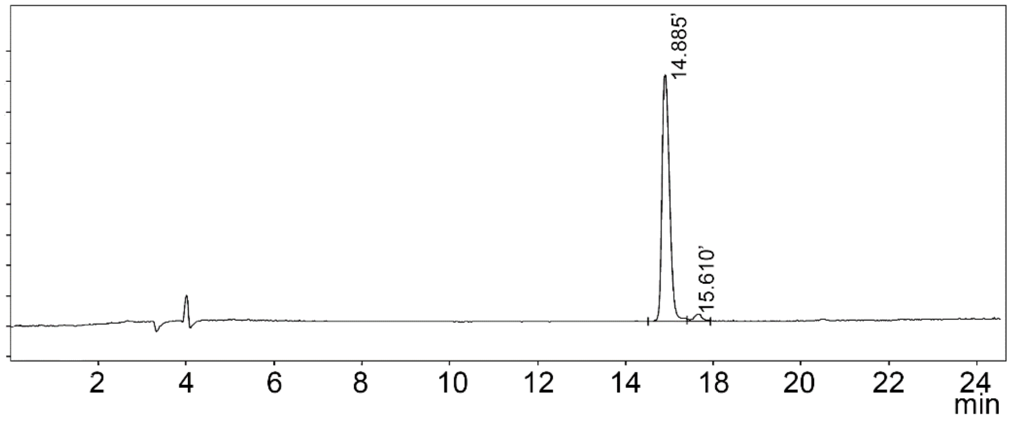

Peak Table

Detector A 220nm

Peak#	Ret. Time	Area	Height	Area%
1	14.885	519925	48198	96.741
2	15.610	17516	1364	3.259
Total	16.512	537441	49562	100.000

Polyphemusin-I Chromatogram

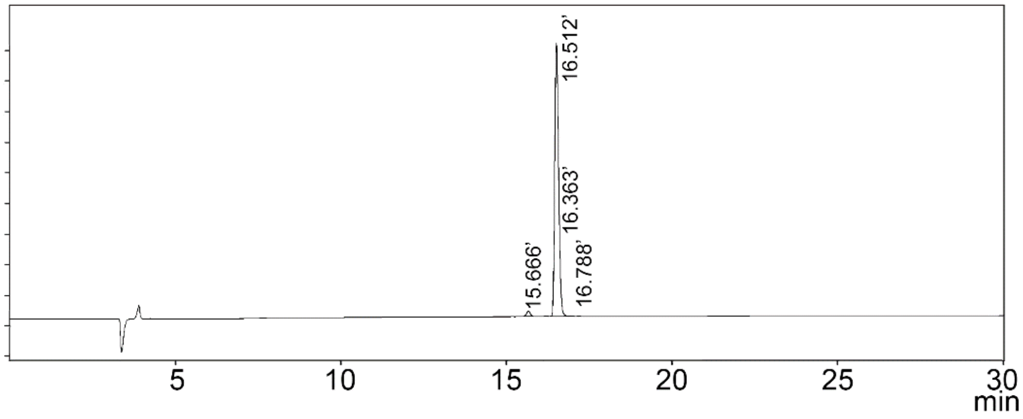

Peak Table

Detector A 214nm

Peak#	Ret. Time	Area	Height	Area%
1	15.666	192433	22249	2.119
2	16.363	26937	2344	0.297
3	16.512	8799340	1082250	96.913
4	16.788	60934	4355	0.671
Total		9079644	1111198	100.000

Supplementary S2. High-performance liquid chromatography for Gallic-Acid-Polyphemusin-I (GAPI) and Polyphemusin-I. The area percentage of the major peak indicated the purity of the testing peptide. The purity percentages of GAPI and polyphemusin-I were 96.74% and 96.91%, respectively.

Gallic-Acid-Polyphemusin-I

Mass Spectrometry Analysis

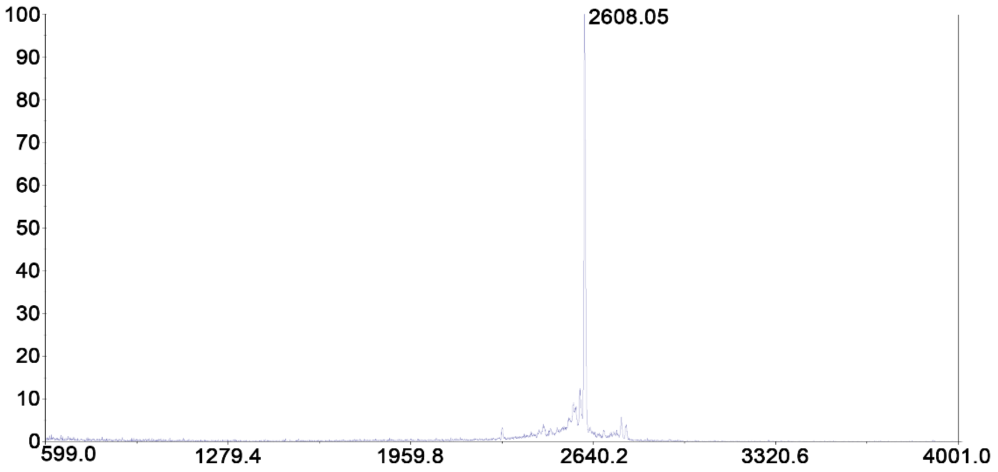

Appearance	Lyophilised material
Molecular Weight (Theoretical)	2607.13
Molecular Weight (by MS, M+H$^+$)	2608.05

Polyphemusin-I

Mass Spectrometry Analysis

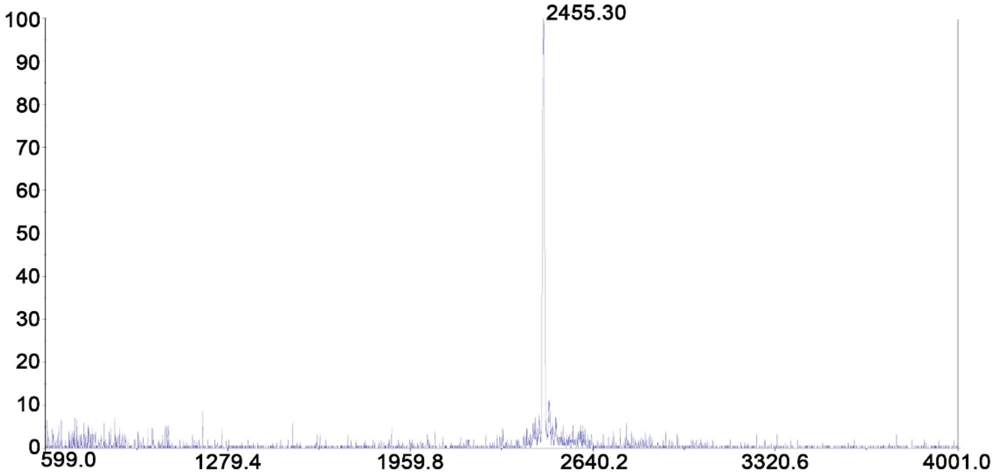

Appearance	Lyophilised material
Molecular Weight (Theoretical)	2455.01
Molecular Weight (by MS, M+H$^+$)	2455.30

Supplementary S3. Mass spectrometry analysis for Gallic-Acid-Polyphemusin-I (GAPI) and Polyphemusin-I. The molecular weight was 2608.05 kD for GAPI and 2455.30 kD for polyphemusin-I. The molecular weight of the synthesised peptide was 152.92 kD more than that of polyphemusin-I. It is equal to the molecular weight of gallic acid minus a water molecule due to polymerisation.

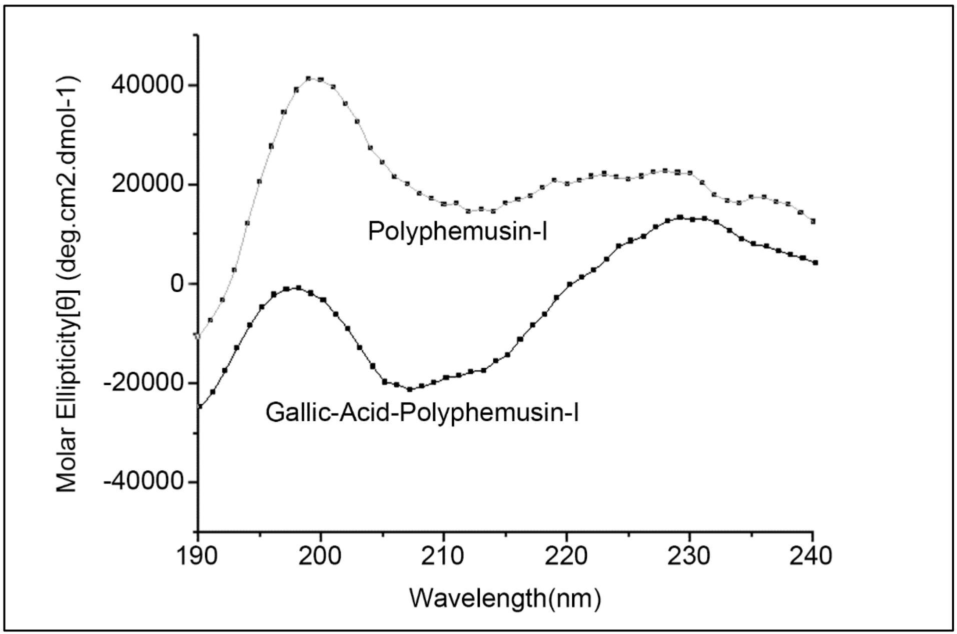

Supplementary S4. Circular dichroism spectroscopy spectrum for for Gallic-Acid-Polyphemusin-I (GAPI) and Polyphemusin-I. The secondary structure was modelled using CDPro software. The secondary structure analysis of GAPI and polyphemusin-I showed that the proportions of the β-sheet were 48.0% and 73.1%, respectively.

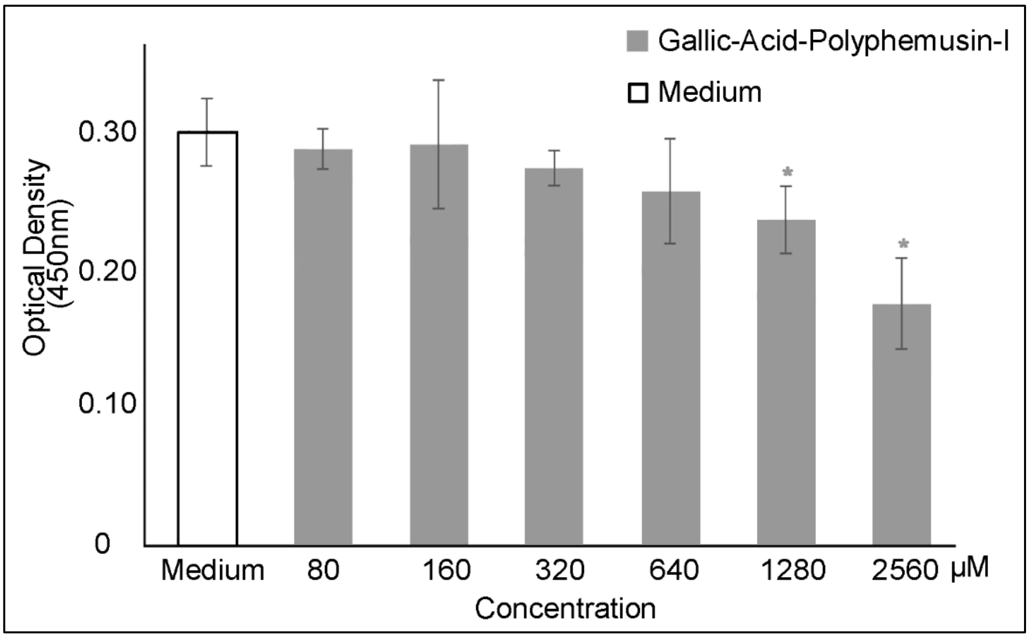

Supplementary S5. Concentration of Gallic-Acid-Polyphemusin-I (GAPI) and optical density values of human gingival fibroblasts. The biocompatibility of GAPI was evaluated by cytotoxicity assay using Cell Counting Kit-8 Assay. Human gingival fibroblast cells were cultured and treated with GAPI. The cells cultured with the only medium were negative controls. The optical density values indicating the number of viable cells was measured at 450 nm after 24 h of incubation. There was no significant difference in optical density values between the 80, 160, 320 and 640 μM GAPI–treated HGF-1 cells and the negative control cell. It revealed that GAPI showed low cytotoxicity to HGF-1 cells.
